# Electroacupuncture for primary insomnia: Protocol for a systematic review and meta-analysis

**DOI:** 10.1097/MD.0000000000011063

**Published:** 2018-07-06

**Authors:** Ziqing Li, Yu Zhang, Yuanping Wang, Xia Yan, Pingchang Xie

**Affiliations:** a The Second Clinical School of Guangzhou University of Chinese Medicine; b Guangdong Provincial Hospital of Chinese Medicine, The Second Clinical School of Guangzhou University of Chinese Medicine, Guangzhou, China.

**Keywords:** electroacupuncture, primary insomnia, protocol, systematic review

## Abstract

**Background:**

Insomnia is a highly widespread sleep disorder in the general population. Electroacupuncture (EA) has been widely received attention as a potential treatment for primary insomnia. However, few previous studies are available to report that EA is a beneficial therapeutic approach to primary insomnia. In addition, there is no critical systematic review or meta-analysis published regarding the effectiveness of this treatment. Here, we provide a protocol to systematically evaluate the efficacy and safety of EA for primary insomnia.

**Methods:**

The reference lists of included studies for relevant randomized controlled trials and 8 electronic databases will be systematically searched by 2 review authors in January 2018, including 4 English databases (PubMed, EMBASE, Cochrane Central Register of Controlled Trials and Cumulative Index to Nursing and Allied Health Literature) and 4 Chinese databases (Chinese National Knowledge Infrastructure, Chinese Biomedical Literature Database, VIP Database, and Wanfang Database). The primary outcomes will be assessed according to the Pittsburgh Sleep Quality Index. Data synthesis will be computed with the use of RevManV5.3 software when a data-analysis is allowed. Methodological quality will be evaluated with the risk of bias according to Cochrane Handbook.

**Results:**

This study will provide a high-quality synthesis of current evidence of EA for primary insomnia.

**Conclusion:**

The conclusion of this systematic review will provide evidence to judge whether EA is an effective therapeutic intervention for patient with primary insomnia.

## Abstract 2

Supplemental Digital Content is available in the text

## 1 Introduction

Insomnia is a highly common and prevalent sleep disorder caused by multiple behavioral, medical, environmental, and psychological factors.^[1]^ It is estimated that 25% to 30% of the adults have occasional sleep difficulties and meet diagnostic criteria of insomnia. About 10% of the patients with severe insomnia suffer from a chronic condition and the symptoms of the condition would last at least several weeks.^[2,3]^ Insomnia is becoming an important factor to influence the health and quality of modern people's life.^[4]^ It may also have a disadvantage effect on study and work, such as decreasing work efficiency, work absenteeism, and work-related accidents.^[5]^ All of these will aggravate the heavy burden of both individuals and society. Furthermore, recent studies have found that insomnia is also associated with higher risk of various cardiovascular disease, cerebrovascular disease, hypertension, and depression.^[6,7]^

Currently, the treatment for insomnia involves various kinds of therapeutic approaches, such as medication, psychotherapy, physical therapy, etc., and the psychological and behavioral therapies are preferred among them.^[8,9]^ One of the most common treatment for insomnia is cognitive behavioral therapy, which is effective to improve the quality of sleep according to cognitive behavioral therapy for insomnia.^[10]^ However, it has not been widely applied in clinical practice as an effective treatment for insomnia owing to poor patients compliance of medication and limited number of trained practitioners.^[11]^ Drugs such as benzodiazepine are thought to be effective for improving sleeping quality.^[12]^ Yet there are potential risks and barriers, including a long-term usage and a range of undesirable side-effects such as memory and performance impairment. Hence, there are an increasing number of insomniacs who are seeking remarkably curative with less adverse effects for primary insomnia.^[13]^

There is a growing need for effective therapy in the field of sleep management and the therapeutic interventions such as acupuncture, electroacupuncture (EA) are widely applied in the treatment for insomnia.^[14]^ Acupuncture is in widespread use and recently several clinical studies and systematic reviews have indicated that it can significantly enhance the effects on the function of treating insomnia.^[15]^ EA is a modified method of conventional acupuncture, which supplies a sequential physical electrical stimulation by inserting acupuncture needles connected to a microcurrent stimulator. Since the body tissue is a kind of electrical conductor, it is believed that the electrical impulses could reinforce the stimulation through the needles at acupoints. Several reported studies have also shown that EA can be efficient for treating insomnia.^[16,17]^

EA has been used in various clinical symptoms, including primary insomnia. The mechanisms behind the effect of EA, however, have not yet been clarified. A study reported that EA is a safe and effective treatment for insomnia and can promote the sleep quality by direct manipulation of autonomic nervous system.^[18]^ In addition, insomnia has been shown its relation to the changes in melatonin and cortisol levels, and a few previous studies has reported the efficacy and safety with the use of melatonin to treat insomnia.^[19,20]^ Previous study has assessed the 24-hour urinary melatonin metabolite rhythm and has suggested that acupuncturing at the H7 acupoint may include regulation of melatonin.^[21]^ Recent studies in animal models have reported that HT7 and GV20 EA stimulation may suppress the secretion of cortisol.^[22,23]^

However, to our knowledge, the randomized controlled trials (RCTs) examining the effectiveness and safety of EA for primary insomnia have never been systematically summarized. As a result, this review will provide a comprehensive analysis of the effects of EA for primary insomnia. The aim to present systemic review is to critically evaluate whether EA is a more effective and safer therapy for primary insomnia with less side-effects compared with traditional therapy.

The objective is to systematically evaluate the effectiveness and safety of EA therapy for patients with primary insomnia.

## 2 Methods

### 2.1 Inclusion criteria for study selection

#### 2.1.1 Types of studies

All the RCTs of EA therapy for primary insomnia will be included. Nonrandomized clinical studies, quasi-RCTs, cluster RCTs, and case studies will be excluded. No writing language or publication types restriction will be applied in this study.

#### 2.1.2 Types of patients

Trials involving participants with primary insomnia will be included without limitations of age, gender, education status, or ethnic background. Primary insomnia should be diagnosed by clinicians based on the Statistical Manual of Mental Disorders—4th Edition criteria.

#### 2.1.3 Types of interventions

The therapeutic intervention applied in the experimental group is EA. Studies using EA in experimental group will be included regardless of the treatment length and frequency. The controlled group can be blank control, placebo, psychological control, or drug therapy (such as benzodiazepines). EA combined with other therapies will be included as well if the combined therapy has the both same groups.

#### 2.1.4 Types of outcome measures

##### 2.1.4.1 Primary outcomes

The Pittsburgh Sleep Quality Index is widely used to evaluate one's sleep quality. It is comprised of 19 self-rated items and 5 other-rated items. The score will indicate the level of sleep quality and the severity of sleep disorders.^[24]^

##### 2.1.4.2 Secondary outcomes

- The Insomnia Severity Index
- Athens Insomnia Scale
- Sleep parameters measured by either subjective or objective approaches, such as actigraphy, polysomnogram, and electroencephalogram
- Adverse effect, such as vomiting, nausea, or dizziness

### 2.2 Search methods for the identification of studies

#### 2.2.1 Electronic searches

Two researchers (YZ and YW) will independently and electronically search 4 English databases (PubMed, EMBASE, Cochrane Central Register of Controlled Trials, and Cumulative Index to Nursing and Allied Health Literature) and 4 Chinese databases (Chinese National Knowledge Infrastructure, Chinese Biomedical Literature Database, VIP Database, and Wanfang Database) from their inception to January 2018. The search items will be used as follows: insomnia, EA. The equivalent search terms will be used in the Chinese databases. The detailed search strategy in PubMed database will be shown in Appendix A in supplementary material and will be modified by searching other databases. The search strategy for PubMed is shown in Appendix A in supplementary material.

#### 2.2.2 Searching other resources

Meta-analysis of the RCTs and relevant systematic reviews will be electronically searched. In addition, reference list of potentially eligible studies and relevant conference proceedings will be manually searched as well to avoid the eligible trials.

### 2.3 Data collection and analysis

#### 2.3.1 Selection of studies

**Figure 1:**
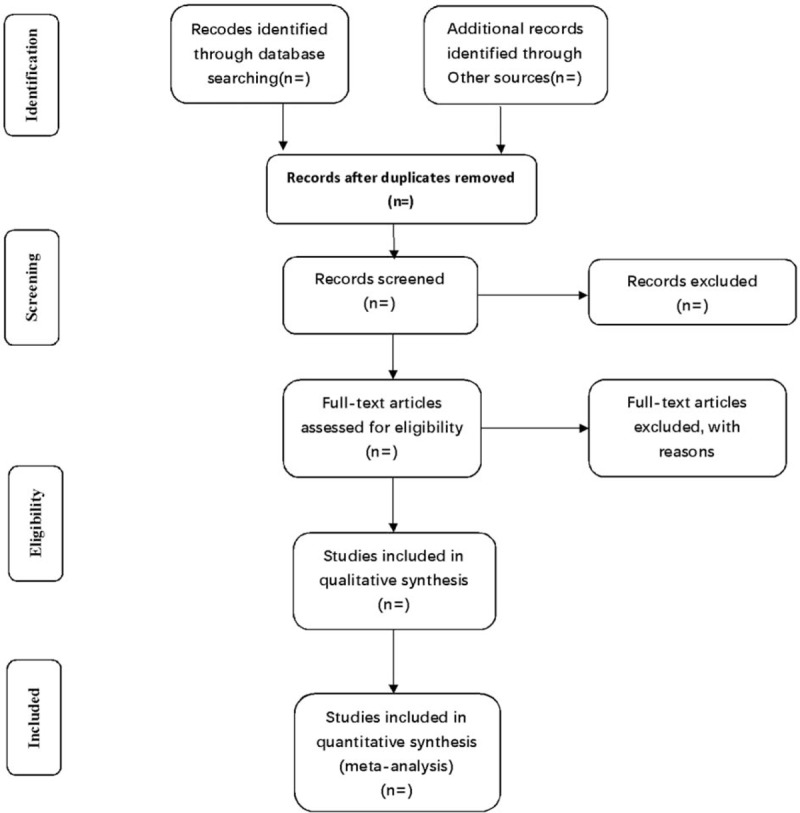
Flow diagram of study selection process.

All reviewers will receive professional training to get familiar with the background, purpose, and process of the review. Relevant studies obtained from the databases mentioned above will be uploaded to the literature management system of EndnoteX7. Two review authors (YZ and YW) will independently perform the selection and record their decisions on a standard eligibility form by screening the titles, abstracts, and key words of the searched articles. Any disagreement about the inclusion of the studies will be resolved through discussion between the 2 review authors. If the discussion cannot reach an agreement, the arbiter will make a final decision of the study selection. Details of the selection procedure for studies are shown in a PRISMA flow chart (Fig. 1).

#### 2.3.2 Data collection and management

Two review authors (YZ and YW) will read all the included articles in full and independently extract the data via a standardized eligibility form. The general information of the selected articles will be extracted, including first author, country, year of publication, study design, duration of follow-up, duration of disease, sample size, detailed intervention, and control treatment. Outcome measures and further information including results, adverse events, and conflicts of interest will be systematically extracted as well. Any disagreement regarding to the data extraction will be discussed and judged between the 2 authors. The results of the data extraction will be checked by the arbiter. When the data of articles are sufficient or ambiguous, one of the authors will contact the original author to request detailed and additional information by e-mail or telephone.

#### 2.3.3 Assessment of risk of bias in included studies

Two review authors (YZ and YW) will independently evaluate the risk of bias of the included articles with the use of Cochrane Handbook V.5.1.0, which includes the following 7 domains: randomized sequence generation, allocation concealment, blinding of participants, personnel and outcome assessors, incomplete outcome data addressed, selective reporting, and other issue. The evaluated domains will be categorized into 3 levers: low risk of bias, high risk of bias, and unclear risk of bias. Any discrepancies will be discussed between the 2 reviews author to reach an agreement. If necessary, a third review author (ZL) will be consulted.

#### 2.3.4 Measures of treatment effect

For continuous data, a standard mean difference with 95% confidence interval (CI) will be used to evaluate the extracted data. For dichotomous outcomes, a rate ratio (RR) with 95% CIs will be used to measure the treatment effect.

#### 2.3.5 Unit of analysis issue

We will only use the data from meta-analysis and randomized studies. If the cross-over trials are included, we will use the first-phase data. If the unit of analysis issue has multiple time point observations, we will categorize the data into 2 terms: a short-term (within 4 weeks) and a long-term (over 4 weeks).

#### 2.3.6 Dealing with missing data

If possible, we will try to contact the first author by e-mail or telephone to request the inadequate and missing data. If we are unable to contact the first author and obtain the missing data, the analysis will only rely on the available data and potential impact of the missing data in the discussion.

#### 2.3.7 Assessment of heterogeneity

According to the guideline of Cochrane Handbook, heterogeneity can be assessed with the chi-squared test. If an I^2^ value exceeds 50%, it will be considered that the heterogeneity among trials is significant. Subgroup analysis will be conducted to explore the potential causes of heterogeneity.

#### 2.3.8 Assessment of reporting biases

If sufficient studies are included in the review (more than 10 trials), visual asymmetry on a funnel plot will be used to detect reporting bias and the test of Egger regression will be used to determine the funnel plot asymmetry.

#### 2.3.9 Data synthesis

RevMan software V5.3 from Cochrane Collaboration will be employed to compute the data synthesis when a meta-analysis is proved suitable. A fixed-effects model will be applied to calculate the RR and mean difference with low heterogeneity (I^2^ < 50%). If not, a random-effects model will be conducted to synthesize the data.

#### 2.3.10 Subgroup analysis

According to the difference in interventions, participant characteristic, controls, and outcome measures, subgroup analysis will be conducted if the number of the included trials is sufficient (at least 10 trials). Subgroup analysis is performed to explore the possible causes of the heterogeneity.

#### 2.3.11 Sensitivity analysis

We will carry out sensitivity analysis to identify the quality and robustness of studies according to the following criteria: methodological quality; sample size; and analysis issue (such as the effect of missing data).

#### 2.3.12 Dissemination and ethics

This systemic review does not need ethical approval because there are no data used in our study that are linked to individual patient data. The findings of this systemic review will provide implication of the effectiveness of EA for primary insomnia. The systematic review will be disseminated in a peer-reviewed journal and published at conference presentations.

#### 2.3.13 Grading the quality of evidence (summary of evidence)

The quality of primary outcome will be evaluated by the Grading of Recommendations Assessment, Development, and Evaluation. The evaluation will divide into 4 levers: high, moderate, low, or very low.

## 3 Discussion

Insomnia has been identified as one of the most common condition comorbid to insomnia and depressive disorders.^[25]^ First-line drugs including benzodiazepines and benzodiazepine receptor agonists are widely applied nowadays. However, these drugs are associated with a lot of undesirable side-effects in long-term treatment, such as leading to high risk of accidents and high rate of mortality.^[26,27]^

EA has been applied in various clinical conditions, including insomnia and psychiatric disorders. It may be an effective treatment for insomnia, and it is unlikely to associate with several side-effects. To the best of our knowledge, the mechanisms underlying the effect of EA for primary insomnia have not been clearly elucidated yet. Therefore, it is necessary to perform a high-quality systematic review and meta-analysis of it, and the process of this review will be shown in the diagram. It is expected that this review can provide rigorous and objective evidences of the effect and safety of EA for primary insomnia. However, there are limitations in this systematic review that may affect the drawn conclusion. First, the included trials are restricted to the publication of English or Chinese, which may limit the search for potential studies. Second, different age of participants and degree of insomnia may run risk of heterogeneity.

## Author contributions

Ziqing Li and Pingchang Xie contributed to the conception of the study. The manuscript of the protocol was drafted by Ziqing Li and was revised by Yu Zhang and Pingchang Xie. The search strategy was developed by all authors and run by Yu Zhang and Pingchang Xie, who will also independently screen the potential studies, extract data of included studies, assess the risk of bias and finish data synthesis. Ziqing Li will arbitrate the disagreements and ensure that no errors occur during the study. All authors have approved the publication of the protocol.

**Conceptualization:** Pingchang Xie, Ziqing Li, Xia Yan.

**Data curation:** Yu Zhang, Ziqing Li, Yuanping Wang, Xia Yan.

**Formal analysis:** Ziqing Li, Yuanping Wang.

**Funding acquisition:** Ziqing Li.

## Supplementary Material

Supplemental Digital Content
