# Supplementary material for: Electroacupuncture for primary insomnia: Protocol for a systematic review and meta-analysis
Source: Medicine (Baltimore). 2018 Jul 6;97(27):e11063. doi: 10.1097/MD.0000000000011063 (PMC6076172; doi:10.1097/MD.0000000000011063)
Supplement: Supplemental Digital Content [file medi-97-e11063-s001.docx]

**Appendix A.**

***Search strategy used in PubMed database***

#1 Disorders of Initiating and Maintaining Sleep OR DIMS (Disorders of Initiating and Maintaining Sleep) OR Early Awakening OR Awakening, Early OR Nonorganic Insomnia OR Insomnia, Nonorganic OR Primary Insomnia OR Insomnia, Primary OR Transient Insomnia OR Insomnia, Transient OR Rebound Insomnia OR Insomnia, Rebound OR Secondary Insomnia OR Insomnia, Secondary OR Sleep Initiation Dysfunction OR Dysfunction, Sleep Initiation OR Dysfunctions, Sleep Initiation OR Sleep Initiation Dysfunctions OR Sleeplessness OR Insomnia Disorder OR Insomnia Disorders OR Insomnia OR Insomnias OR Chronic Insomnia OR Insomnia, Chronic OR Psychophysiological Insomnia OR Insomnia, Psychophysiological

#2 Electroacupuncture OR Electro-acupuncture

#3 Randomized controlled trial OR clinical study OR Clin-ical Trial OR Controlled study OR Controlled Trial OR Random*Control* study OR random* Control* Trial

#1 AND #2 AND #3
